# Cardiac assessments of bottlenose dolphins (*Tursiops truncatus*) in the Northern Gulf of Mexico following exposure to *Deepwater Horizon* oil

**DOI:** 10.1371/journal.pone.0261112

**Published:** 2021-12-14

**Authors:** Barbara K. Linnehan, Forrest M. Gomez, Sharon M. Huston, Adonia Hsu, Ryan Takeshita, Kathleen M. Colegrove, Craig A. Harms, Ashley Barratclough, Alissa C. Deming, Teri K. Rowles, Whitney B. Musser, Eric S. Zolman, Randall S. Wells, Eric D. Jensen, Lori H. Schwacke, Cynthia R. Smith

**Affiliations:** 1 National Marine Mammal Foundation, San Diego, California, United States of America; 2 San Diego Veterinary Cardiology, San Diego, California, United States of America; 3 Zoological Pathology Program, University of Illinois at Urbana-Champaign, Brookfield, Illinois, United States of America; 4 North Carolina State University, Center for Marine Sciences and Technology, Morehead City, North Carolina, United States of America; 5 Dauphin Island Sea Lab, Dauphin Island, Alabama, United States of America; 6 National Oceanic and Atmospheric Administration, Office of Protected Resources, Silver Spring, Maryland, United States of America; 7 Chicago Zoological Society’s Sarasota Dolphin Research Program, c/o Mote Marine Laboratory, Sarasota, Florida, United States of America; 8 U.S. Navy Marine Mammal Program, Naval Information Warfare Center Pacific, San Diego, California, United States of America; University of Siena, ITALY

## Abstract

The *Deepwater Horizon* (DWH) oil spill profoundly impacted the health of bottlenose dolphins (*Tursiops truncatus*) in Barataria Bay, LA (BB). To comprehensively assess the cardiac health of dolphins living within the DWH oil spill footprint, techniques for in-water cardiac evaluation were refined with dolphins cared for by the U.S. Navy Marine Mammal Program in 2018 and applied to free-ranging bottlenose dolphins in BB (*n* = 34) and Sarasota Bay, Florida (SB) (*n* = 19), a non-oiled reference population. Cardiac auscultation detected systolic murmurs in the majority of dolphins from both sites (88% BB, 89% SB) and echocardiography showed most of the murmurs were innocent flow murmurs attributed to elevated blood flow velocity [1]. Telemetric six-lead electrocardiography detected arrhythmias in BB dolphins (43%) and SB dolphins (31%), all of which were considered low to moderate risk for adverse cardiac events. Echocardiography showed BB dolphins had thinner left ventricular walls, with significant differences in intraventricular septum thickness at the end of diastole (*p* = 0.002), and left ventricular posterior wall thickness at the end of diastole (*p* = 0.033). BB dolphins also had smaller left atrial size (*p* = 0.004), higher prevalence of tricuspid valve prolapse (*p* = 0.003), higher prevalence of tricuspid valve thickening (*p* = 0.033), and higher prevalence of aortic valve thickening (*p* = 0.008). Two dolphins in BB were diagnosed with pulmonary arterial hypertension based on Doppler echocardiography-derived estimates and supporting echocardiographic findings. Histopathology of dolphins who stranded within the DWH oil spill footprint showed a significantly higher prevalence of myocardial fibrosis (*p* = 0.003), regardless of age, compared to dolphins outside the oil spill footprint. In conclusion, there were substantial cardiac abnormalities identified in BB dolphins which may be related to DWH oil exposure, however, future work is needed to rule out other hypotheses and further elucidate the connection between oil exposure, pulmonary disease, and the observed cardiac abnormalities.

## Introduction

The 2010 *Deepwater Horizon* (DWH) oil spill affected ecologically important organisms throughout all trophic levels. Novel research in the wake of the spill has elucidated pathophysiological mechanisms for cardiotoxic effects of DWH oil in multiple animal species, especially those driven by polycyclic aromatic hydrocarbons (PAHs). Crude oil components impair cardiac excitation-contraction coupling in fish, prolonging the action potential of cardiomyocytes [2]. In fish embryos, crude oil exposure causes direct developmental toxicity to the developing heart, with dose-dependent effects on cardiac function resulting in circulatory disruption, pericardial edema, and other malformations [3, 4]. This response to oil is highly conserved across fish species [3, 4]. Acute exposure can also have cardiotoxic effects, as demonstrated in young adult mahi-mahi, where exposure to PAHs for 24 hours caused reduced stroke volume and cardiac output [5]. One hypothesis proposes that cardiotoxicity is driven by the PAH phenanthrene disrupting the physiology of heart muscle cells, and the cellular targets for this toxic compound are highly conserved across vertebrates [6]. Additionally, the combined effects of elevated temperature and DWH oil exposure have been demonstrated to cause dose-dependent impairments and functional heart failure of larval mahi-mahi [7].

In a laboratory study with rats, inhalation of crude oil vapor negatively impacted blood pressure and responsiveness of the heart to an inotropic drug [8]. These effects were seen one day post-exposure and the effects resolved after 28 days post-exposure. In birds, double-crested cormorants with low to moderate dermal exposure to weathered DWH oil resulted in gross morphological changes including softer, more distensible ventricular walls, and echocardiographic changes of decreased ventricular myocardial contractility, as well as arrhythmias on electrocardiogram (ECG) [9].

Human studies have also reported cardiotoxic effects associated with DWH oil exposure. People who lived in close proximity to the spill or were involved in cleanup operations had a significantly increased risk (29–43%) of heart disease events, including non-fatal heart attacks within 5 years following the oil spill [10, 11]. Additionally, people who worked on the spill for >180 days and who experienced heat stress had an increased risk of nonfatal myocardial infarction within 1 to 3 years post-spill [10]. In a follow-up study, oil spill workers with estimated maximum total hydrocarbon exposure levels of ≥0.3 ppm showed 62–81% higher hazards for heart attack 5 years after the spill compared to workers with lower hydrocarbon exposures [12, 13]. In addition to heart attacks, abnormal cardiac function in workers involved in the oil spill cleanup included abnormal ECGs (ventricular conduction delays, anterior fascicular blocks, sinus rhythm nonspecific T waves, sinus bradycardia ST and T waves, and sinus rhythm early repolarizations) and ventricular hypertrophy, which persisted in many workers 7 years after the oil spill [14].

Bottlenose dolphins (*Tursiops truncatus*) in Barataria Bay, Louisiana (BB) suffered a variety of adverse health effects related to DWH oil exposure, including pulmonary, reproductive, and endocrine disease, changes in immune function, and reduced survival rate [15–23]. The effects of oil on cardiac health in this species, or other cetaceans, had not been previously examined. Given the consistency of cardiotoxic effects observed across other vertebrate species, and the potential impacts on cetacean survival, there was a strong need to comprehensively assess cardiac health and survey for evidence of cardiotoxic effects in this population.

Dolphin cardiology is still in its infancy, and cardiac evaluation has long been a data gap in the health assessments of free-ranging dolphins. There were no baseline cardiac data on the BB dolphin population prior to the DWH spill, and there is a paucity of information about dolphin cardiac health in general. Recent studies have laid the foundation for cardiac assessment techniques in cetaceans, including electrocardiography (ECG) in managed and free-ranging cetaceans [24–29] and echocardiography in dolphins managed under human care [30–33]. In this study, techniques for advanced cardiac assessment were refined with managed care dolphins, then applied to free-ranging dolphins in BB and dolphins outside of the oil spill footprint in SB. Cardiac auscultation and the prevalence and characteristics of murmurs in BB dolphins were described in Linnehan *et al*. [1]; the results presented here were collected during the same health assessments and from the same group of dolphins as in the auscultation study. Additionally, postmortem exams and histopathology were performed on dolphins stranded within the oil spill footprint and were compared to dolphins stranded outside of the oil spill footprint. The goal of the current study was to comprehensively assess the cardiovascular health of free-ranging dolphins in the northern Gulf of Mexico oil spill footprint, using echocardiography, ECG, and histopathology.

## Materials and methods

### Animals

For echocardiogram technique refinement specific to in-water applications, 15 bottlenose dolphins cared for by the U.S. Navy Marine Mammal Program (Navy) in San Diego, California, participated in echocardiograms from March 2018 to August 2019. Navy dolphin participation was approved by the Navy Marine Mammal Program’s Institutional Animal Care and Use Committee (NIWC Pacific IACUC # 128–2018) and from the U.S. Navy Bureau of Medicine and Surgery (NRD-1157). The Navy Marine Mammal Program is accredited by the Association for Assessment and Accreditation of Laboratory Animal Care International and adheres to the national standards of the U.S. Public Health Service Policy on the Humane Care and Use of Laboratory Animals and the Animal Welfare Act.

Free-ranging bottlenose dolphins were examined during catch-and-release health assessments in Sarasota Bay, FL (SB) in June 2018, a non-oiled reference location, under National Marine Fisheries Service (NMFS) Scientific Research Permit No. 20455. All dolphins in this study were given complete veterinary examinations as previously described [19, 34]. Nineteen dolphins were examined in SB, 10 males and 9 females (1 confirmed pregnant on ultrasound), ranging in age from 2 to 47 years, and 168 to 281cm in total length; 17 of these dolphins received echocardiograms. The SB population is a particularly valuable reference group due to the long-term research on health, ecology, behavior, life history, and human interactions [35]. Catch-and-release health assessments were performed in BB in July 2018 under NMFS Scientific Research Permit No. 18786. Thirty-four dolphins were examined in BB, including 21 males and 13 females (6 confirmed pregnant on ultrasound), ranging from 195 to 267 cm in total length; 29 of these dolphins received echocardiograms. The methods of temporary capture and on-site release in both locations followed those previously described [19, 34, 35].

### Transthoracic echocardiography

All transthoracic echocardiograms were performed by board-certified veterinary cardiologists (SH or AH) using the GE Vivid-iq ultrasound and GE 3S phased-array transducer cardiac probe (1.5 to 3.6 MHz) (GE Healthcare, Chicago, IL, USA). Electrical tape was used to cover the cord and sides of the probe for additional waterproofing. Ultrasound goggles were worn by the examining cardiologists (FP View 3D, iTVGoggles, Los Angeles, CA, USA), covered with a cloth hood to help with image visualization in bright sunlight. The following standard echocardiogram images were examined: right parasternal long-axis, right parasternal short-axis, left parasternal apical, left parasternal cranial long-axis, and left parasternal short-axis.

Each valve was interrogated with color Doppler. Regurgitant jets were classified subjectively as none (absent), trace, small, medium, or large. Classification criteria were defined by the cardiologists based on standard evaluation of color flow jet origin, direction, and size [36]. Trace regurgitation was defined as a barely detectable jet noted on color Doppler (a few color pixels). A small jet was defined as relatively narrow, not distending very far from the valve being interrogated, not persisting throughout the entirety of systole and/or appearing only mildly turbulent based on the mosaic color flow pattern. A medium jet was defined as one that appeared turbulent within at least ¼ of the associated chamber. A large jet was defined as one that appeared turbulent within more than ½ of the associated chamber.

When valvular regurgitation was identified, the peak regurgitant flow velocity was measured using continuous wave Doppler. When applicable, pulmonary arterial pressures were non-invasively estimated with echocardiography using Doppler analysis for tricuspid and pulmonary valvular flow to detect regurgitant jets according to previously established techniques [37–41]. The maximum pulmonic regurgitant flow velocity was then used in the modified Bernoulli equation to calculate the estimated pressure gradient across the pulmonic valve from the pulmonary artery to the right ventricle. The maximum tricuspid valve regurgitant flow was used to calculate the systolic pressure gradient between the right atrium and ventricle. Systolic pulmonary arterial pressure was calculated by adding the estimated right atrial pressure to the systolic right atrium-ventricle pressure gradient. Standard categories in domestic animals were utilized, with mild pulmonary hypertension defined as pulmonary arterial pressure 30-50mmHg, moderate 51-75mmHg, and severe >75mmHg [39].

In order to normalize for differences related to sex, age, and size of the dolphins (to allow for comparisons), each dolphin’s cardiac measurements were indexed to his/her aortic diameter measurement (Ao). Previous studies in humans have demonstrated that age, height, weight, and sex are the principal determinants of aortic diameter dimensions, with males and older individuals having larger aortas [42].

#### Navy dolphins

**Fig 1:**
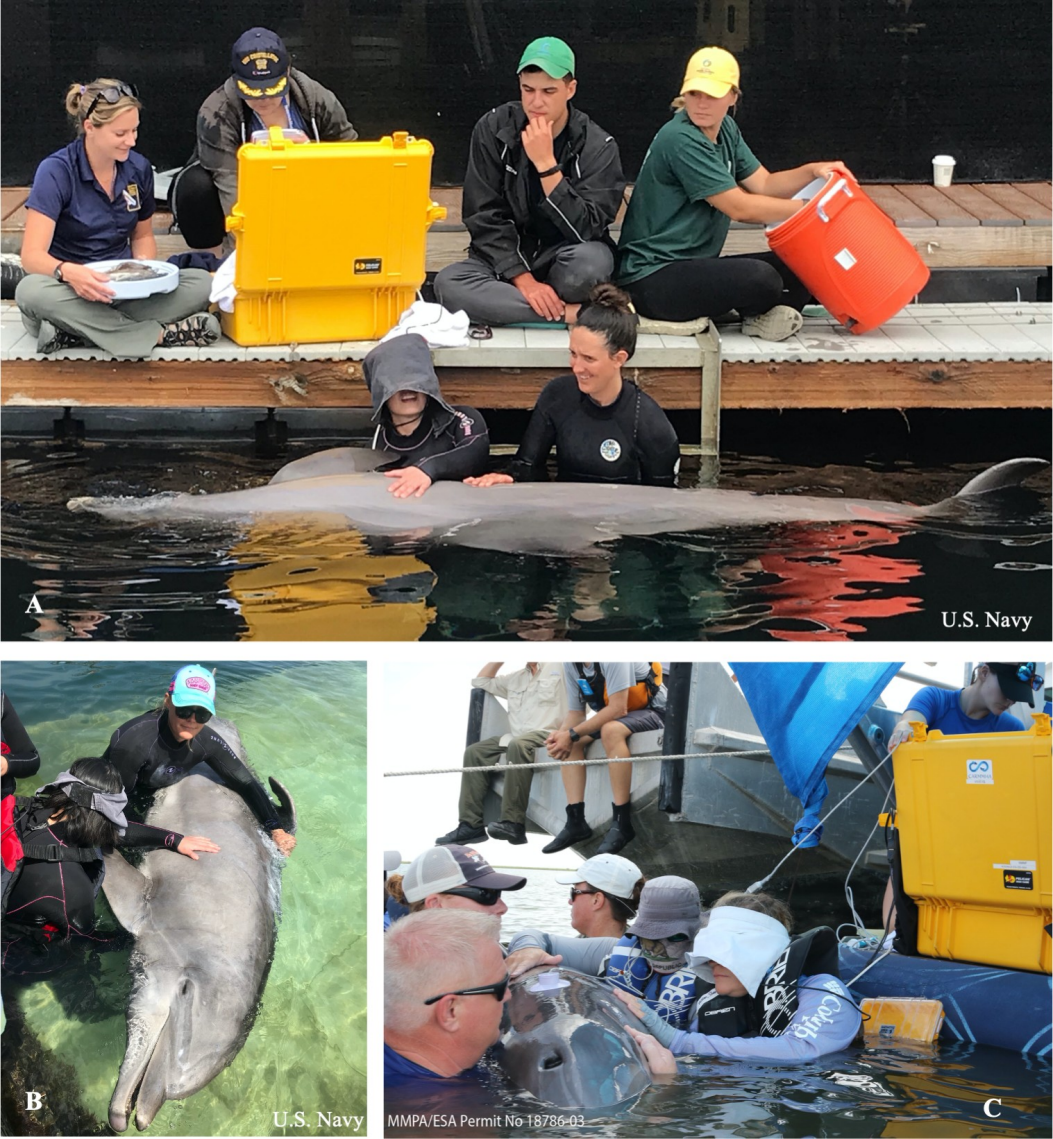
In-water transthoracic echocardiograms. (A) Navy dolphin voluntarily participating for a transthoracic echocardiogram in a lateral presentation. The trainer and cardiologist stand on a submerged platform (A) or medical platform (B). (Republished from the U.S. Navy under a CC BY license, with permission from U.S. Navy, original copyright 2021.) (C) Transthoracic echocardiogram being performed on a dolphin in BB, while the animal is gently restrained in an obliqued orientation beside a boat.

Echocardiograms were performed with Navy dolphins to aid in technique refinement for in-water dolphin applications. Twenty-three in-water transthoracic echocardiograms were performed with 15 Navy dolphins. Using positive reinforcement training techniques, dolphins assumed a dorsal presentation, lateral recumbency, or 45-degree lateral recumbency with a trainer standing on a submerged platform (Fig 1A and 1B). The cardiologist stood on the platform beside the trainer to facilitate scanning of the cardiac window in one or all of these three positions.

#### Free-ranging dolphins

In SB, 17 dolphins received in-water transthoracic echocardiograms. In BB, 29 dolphins received in-water transthoracic echocardiograms. Dolphins were gently restrained in-water by experienced handlers, keeping the dolphin mostly submerged with the blowhole above the surface. Echocardiograms were performed by cardiologists while standing beside the dolphin and reaching under the body to access the cardiac window. This was typically performed next to a boat, so that electronic equipment could stay dry and shaded onboard (Fig 1C). Dolphins were examined in dorsal presentation or in a 45-degree tilt to the right or left to allow for better visualization of the cardiac window. Most exams were completed in 5–7 minutes.

### Transesophageal echocardiography

All transesophageal echocardiograms were performed using the GE Vivid-iq ultrasound and GE 6VT-D TEE ultrasound transducer (3-8MHz). Electrical tape and Parafilm^®^ M laboratory film (Bemis Company, Inc., Oshkosh, WI, USA) were wrapped around the probe and cord for additional waterproofing. As with transthoracic exams, ultrasound goggles were worn by the examining cardiologists (FP View 3D, iTVGoggles, Los Angeles, CA, USA), covered with a cloth hood to help with image visualization in bright sunlight.

#### Navy dolphins

During technique development, 11 transesophageal echocardiograms were performed on eight dolphins. Dolphins were gently restrained and the transesophageal probe passed down the esophagus.

#### Free-ranging dolphins

In SB, transesophageal echocardiograms were performed with five dolphins. Dolphins were gently restrained in the water and the transesophageal probe passed down the esophagus.

### Electrocardiograms

**Fig 2:**
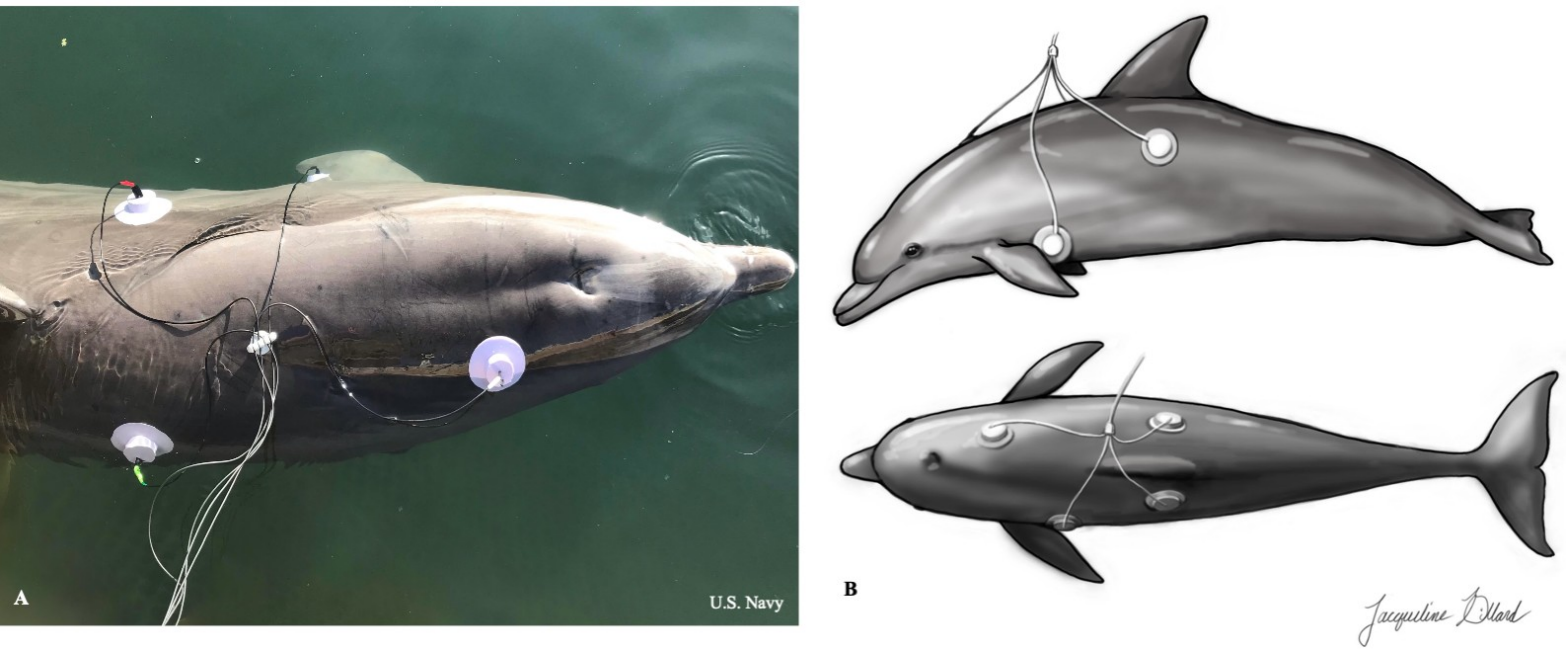
Telemetric four-electrode six-vector ECG with modified base-apex lead placement. (A) A trained Navy dolphin demonstrating the modified base-apex lead placement utilized for telemetric ECG. (Republished from the U.S. Navy under a CC BY license, with permission from U.S. Navy, original copyright 2021.) (B) Illustration of modified base-apex lead placement on a dolphin (illustrated by Jacqueline Dillard).

ECGs were monitored in real time and recorded using a telemetric ECG system (Televet^®^ ECG 100, DVM Solutions, San Antonio TX, USA, and Televet 100 Version 6.0.0 software) with Bluetooth transmission communicating to a laptop (Dell Latitude E7240) or tablet (Microsoft Surface Pro 6) computer. Four-electrode six-vector ECG recordings were acquired using the cables and electrodes supplied with the Televet ECG 100 system and ECG contact gel (Spectra^®^ 360 Electrode Gel, Parker Laboratories, Fairfield, NJ, USA). The system was modified for use in wet and shallow submerged conditions by enclosing the receiver/transmitter unit in a hard plastic water-resistant case modified with a custom cable port, and molding soft silicone gel suction cups around the electrodes (courtesy A. Fahlman). Suction cup attachment reduced electrical noise during out-of-water recordings when water was splashed on the dolphin to keep skin moist, and allowed for in-water recordings. Surface electrode placement was adapted from equine cardiology recommendations [43] as previously reported for bottlenose dolphins [24] based on the primary QRS vectors of cetaceans and other animals with cardiac group B ventricular activation [44]. The right front electrode was placed dorsally above the right scapula over the heart base, the left front electrode was placed ventrally in or near the left axilla over the heart apex, converting lead I into a base-apex lead (Fig 2). The right rear and left rear electrodes are placed on the flanks below the trailing edge of the dorsal fin near the level of the vertebral transverse processes. The base-apex lead typically maximizes the QRS amplitude compared with frontal plane recordings [24]. Recordings of ECGs were evaluated using the Televet 100 software and visually for rate, rhythm and abnormal complexes.

Additionally, ECG was monitored and recorded during echocardiograms using the cables and electrodes connected to the GE Vivid-iq ultrasound machine. The three electrodes were positioned in the same modified base-apex lead placement as above. These leads were also modified for in-water use by molding soft silicone gel suction cups around the electrodes. Three vector leads (I, II, III) could be displayed (one lead at a time) on the GE Viviq-iq ultrasound screen.

### Cardiac auscultation

Cardiac auscultation was systematically performed as previously described with Navy and free-ranging dolphins [1]. All auscultations were performed in seawater with the 3M™ Littman® Veterinary Master Classic II 32” (3M™ Littman®, St. Paul, MN, USA).

Nineteen dolphins were auscultated in SB and 34 in BB by a cardiologist (AH or SMH) in addition to other marine mammal veterinarians (BKL, FMG, CRS) according to standard procedure [1]. Murmurs were graded by intensity using the conventional Grade I-VI scale [45–47].

### Histopathology

All post-mortem examinations of stranded dolphins were conducted by members of the National Oceanic and Atmospheric Administration’s (NOAA) NMFS U.S. Marine Mammal Stranding Network (MMSN) in the field or at participating institution laboratories using standardized protocols. These dolphins were distinct from the live dolphins examined during the catch-and-release health assessments.

#### Northern Gulf of Mexico dolphins (NGoM)

For assessment of heart lesions in stranded dolphins in the oil spill footprint (NGoM), heart samples from 100 bottlenose dolphins with >115 cm standard length that stranded between June 2010 and February 2019 in Louisiana (*n* = 49), Mississippi (*n* = 20), Alabama (*n* = 13), and the Florida Panhandle (*n* = 18) were evaluated. All dolphins had hematoxylin and eosin-stained slides of heart available for histologic evaluation and had tissues not compromised by severe decomposition (Code 1 to 3 carcasses). General pathologic findings from a subset of cases stranding in years immediately following the DWH oil spill were included in a previous publication [16]. For some dolphins, age was evaluated by tooth dentin layer analysis via previously described methods [48–50].

#### Reference dolphins

Hematoxylin and eosin stained slides of heart tissue were available from 42 reference population dolphins >115 cm standard length to compare to lesions noted in dolphins stranding in the oil spill footprint. All dolphins stranded outside of the geographic area and time period of the DWH oil spill, in either North Carolina (*n* = 3; stranding in 2014), Virginia (*n* = 1; stranding in 2015), or along the west coast of Florida (*n* = 38; stranding between 2002–2009 and 2015–2016), and had no known history of oil exposure. Reference dolphins included some dolphins also used as control dolphins for a previous study [16]. Where possible, age was determined via known animal life history and photo-identification records.

#### Heart lesion scoring

Heart tissue (in most cases including left ventricle, right ventricle, interventricular septum, atria and aorta) from NGoM and reference cases were evaluated in a standardized fashion by a single pathologist blind to case ID and stranding location to reduce bias. The following lesions were evaluated in all cases: myocyte nuclear rowing, myocyte nuclear karyomegaly, myocardial fibrosis, myocardial fibrosis severity, myocarditis, myocardial degeneration and/or necrosis, vascular arteriosclerosis, and myocardial lipofuscinosis. Slides were stained with Masson’s trichrome to evaluate interstitial fibrosis. Fibrosis was subjectively graded as mild, moderate, or severe based on the percentage of myocardium affected. Cases diagnosed with heart failure had some or all of the following gross or histologic lesions: thoracic effusion, peritoneal effusion, diffuse pulmonary edema, pulmonary congestion and accumulation of histiocytes within alveolar spaces containing phagocytized red blood cells or hemosiderin, centrilobular congestion, centrilobular fibrosis, and centrilobular hepatocellular atrophy or loss.

### Data and statistical analysis

Echocardiograms were analyzed using GE Vivid-iq ultrasound software, EchoPAC version 201. Both cardiologists reviewed each study and made all qualitative and quantitative measurements. Forty-six morphological and visual parameters were analyzed in addition to 52 measured indices, including 2D, M-mode and spectral Doppler measurements. ECG recordings were analyzed for rhythm, lowest and highest heart rate, presence of arrhythmias, and quality of the recording. The prevalence of cardiac parameters was compared between SB and BB dolphin groups using unpaired two-sample t-tests or two-sided Fisher’s exact tests (R version 3.5.0, The R Foundation for Statistical Computing; package *ggpubr*). To summarize the results of these comparisons, we used *p* ≤ 0.05 as a threshold for significance, but reported all *p*-values for each test performed. Prevalence of heart lesions observed in NGoM dolphins was compared to lesion prevalence in reference dolphins using Fisher’s exact. Tooth age and total body length (as a proxy for age) was compared using Mann-Whitney U tests.

## Results

### Echocardiograms

In both Navy and free-ranging dolphins, poor image quality was obtained via transesophageal echocardiography due to lung interference, except for a brief moment during spontaneous exhalation. Transesophageal echocardiography did not contribute additional information beyond that obtained during transthoracic echocardiography exams, and therefore data for all echocardiographic analyses were taken from transthoracic studies.

Transthoracic echocardiographic exams were performed in SB (*n* = 17) and BB (*n* = 29). Indexing each dolphin’s measurements to aortic diameter allowed for comparisons across dolphins of different sizes. There was no significant difference in aortic diameter by site (*p* = 0.15). Aortic diameter was a strong linear predictor of both weight (*R*^*2*^ = 0.41; *F*(1,28) = 19.5; *p*<0.000) and length (*R*^*2*^ = 0.50; *F*(1,41) = 41.6; *p*<0.000), as expected based on extrapolating from other species. Specific morphometric and visual parameters of interest were selected and then compared between sites (Tables 1 and 2).

**Table 1:** Summary of mean two-dimensional echocardiographic measurements of interest.

Echocardiogram measurement	SB mean ± SD	BB mean ± SD	*p-*value
Fractional shortening (%)	33.24 (± 9.2)	37.04 (± 4.79)	0.141
IVSd (cm)	1.69 (± 0.29)	1.48 (± 0.28)	**0.026**
IVSd/Ao	0.4 (± 0.07)	0.33 (± 0.06)	**0.002**
LA (cm)	5.24 (± 1.09)	5.02 (± 0.76)	0.476
LA/Ao	1.24 (± 0.13)	1.12 (± 0.12)	**0.004**
LVIDd (cm)	7.27 (± 1.46)	7.48 (± 0.68)	0.594
LVIDs (cm)	4.88 (± 1.28)	4.71 (± 0.52)	0.618
LVIDd/Ao	1.7 (± 0.25)	1.68 (± 0.2)	0.728
LVIDs/Ao	1.15 (± 0.25)	1.07 (± 0.14)	0.250
LVPWd (cm)	1.52 (± 0.36)	1.36 (± 0.34)	0.149
LVPWd/Ao	0.36 (± 0.08)	0.3 (± 0.07)	**0.033**
LVPWdelta (cm)	51.31 (± 20.38)	62.15 (± 39.13)	0.240

Abbreviations: IVSd = intraventricular septal thickness in diastole; Ao = aortic diameter; LA = left atrium; LVPWd = left ventricular posterior wall thickness in diastole; LVIDs = left ventricular internal dimension in systole; LVIDd = left ventricular internal dimension in diastole.

Measurements were compared between SB and BB, and *p*-values ≤ 0.05 are reported in bold.

**Table 2:** Summary of echocardiographic findings of interest.

Echocardiogram finding	SB cases	BB cases	SB prevalence (95% CI)	BB prevalence (95% CI)	Relative risk	*p-*value
Aorta dilation	1 / 20	0 / 34	0.05 (0–0.25)	0 (0–0.12)	0	0.370
Aortic valve thickening	0 / 18	10 / 29	0 (0–0.21)	0.34 (0.2–0.53)	NA	**0.008**
Mitral valve prolapse	3 / 18	10 / 29	0.17 (0.05–0.4)	0.34 (0.2–0.53)	2.07	0.315
Mitral valve regurgitation	5 / 20	6 / 34	0.25 (0.11–0.47)	0.18 (0.08–0.34)	0.71	0.728
Pulmonary artery dilation	0 / 16	1 / 23	0 (0–0.23)	0.04 (0–0.23)	NA	1.000
Tricuspid valve prolapse	0 / 15	11 / 27	0 (0–0.24)	0.41 (0.24–0.59)	NA	**0.003**
Tricuspid valve regurgitation	3 / 20	6 / 34	0.15 (0.04–0.37)	0.18 (0.08–0.34)	1.18	1.000
Tricuspid valve thickening	1 / 16	10 / 27	0.06 (0–0.3)	0.37 (0.21–0.56)	5.93	**0.033**

Prevalence was compared between SB and BB, and *p*-values ≤ 0.05 are reported in bold.

**Fig 3:**
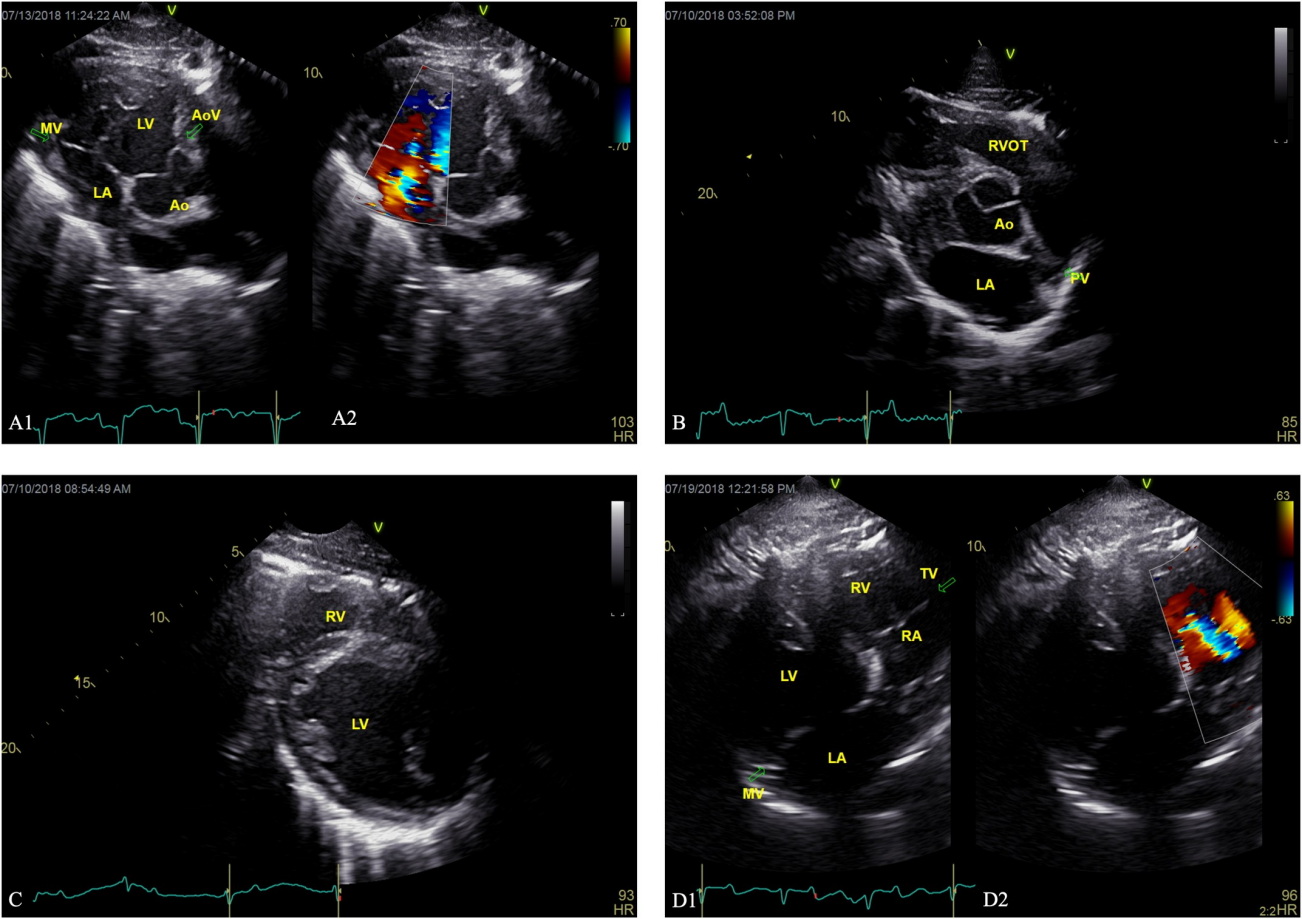
Echocardiogram images from BB dolphins. (A1) Two-dimensional right parasternal short axis view of the mitral valve and (A2) color Doppler showing medium mitral regurgitation in a BB dolphin. (B) Two-dimensional right parasternal short axis view of the left atrium and aorta in a BB dolphin. (C) Two-dimensional right parasternal short axis view of the left ventricle of a BB dolphin with thin left ventricular walls. (D1) Two-dimensional right parasternal short axis view of the tricuspid valve and (D2) color Doppler showing mild tricuspid regurgitation in a BB dolphin. Labels: MV = mitral valve, LV = left ventricle, LA = left atrium, Ao = aorta, AoV = aortic valve, RVOT = right ventricular outflow tract, PV = pulmonic valve, RV = right ventricle, MV = mitral valve, RA = right atrium, TV = tricuspid valve.

The heart structure and size two-dimensional measurements were compared between the two populations, and mean values reported in Table 1. The size of the left ventricular walls was significantly different between BB and SB. In comparing intraventricular septum thickness at the end of diastole (IVSd) and IVSd indexed to aorta, BB dolphins had significantly smaller IVSd than SB dolphins, indicating thinner walls (IVSd, *p* = 0.026, IVSd/Ao, *p* = 0.002) (Fig 3). Comparing left ventricular posterior wall thickness at the end of diastole (LVPWd) indexed to aorta, BB dolphins had significantly thinner walls (LVPWd/Ao, *p* = 0.033). In addition, BB dolphins had significantly smaller left atria than SB dolphins when comparing the ratio of LA/Ao between sites (*p* = 0.004) (Table 1, Fig 3).

Valve morphology and function were analyzed and compared between sites (Table 2). One dolphin in SB and two dolphins in BB had medium mitral regurgitation based on color flow Doppler signal appearance. The rest of the dolphins in both locations had trace or small regurgitant jets across one or more valves (*n* = 39); trace or small regurgitation is considered a variation of normal and not clinically significant [36, 51]. Five dolphins had no regurgitation or insufficiency (Table 3). There was a significantly higher prevalence of tricuspid valve prolapse in BB vs SB (*p* = 0.003). There was a significant difference in tricuspid valve structure, with BB having a higher prevalence of tricuspid valve thickening compared to SB (*p* = 0.033). Additionally, there was a significant difference in aortic valve structure between the two sites, with BB having a higher prevalence of mild aortic valve thickening (*p* = 0.008).

**Table 3:** Abnormal valvular regurgitation and insufficiency findings from echocardiographic analyses of BB dolphins.

	Regurgitation finding					
	None	Trace	Small jet	Medium jet	Pre-systolic	Not assessed*
Mitral valve	18	16	8	3	1	1
Tricuspid	17	13	9	0	3	5
	Insufficiency finding					
	None	Trace	Mild	Medium	Severe	Not assessed*
Aortic	32	11	1	0	0	3
Pulmonic	19	8	0	0	0	20

* No assessment was conducted if the color flow was not well visualized/was inadequate.

**Fig 4:**
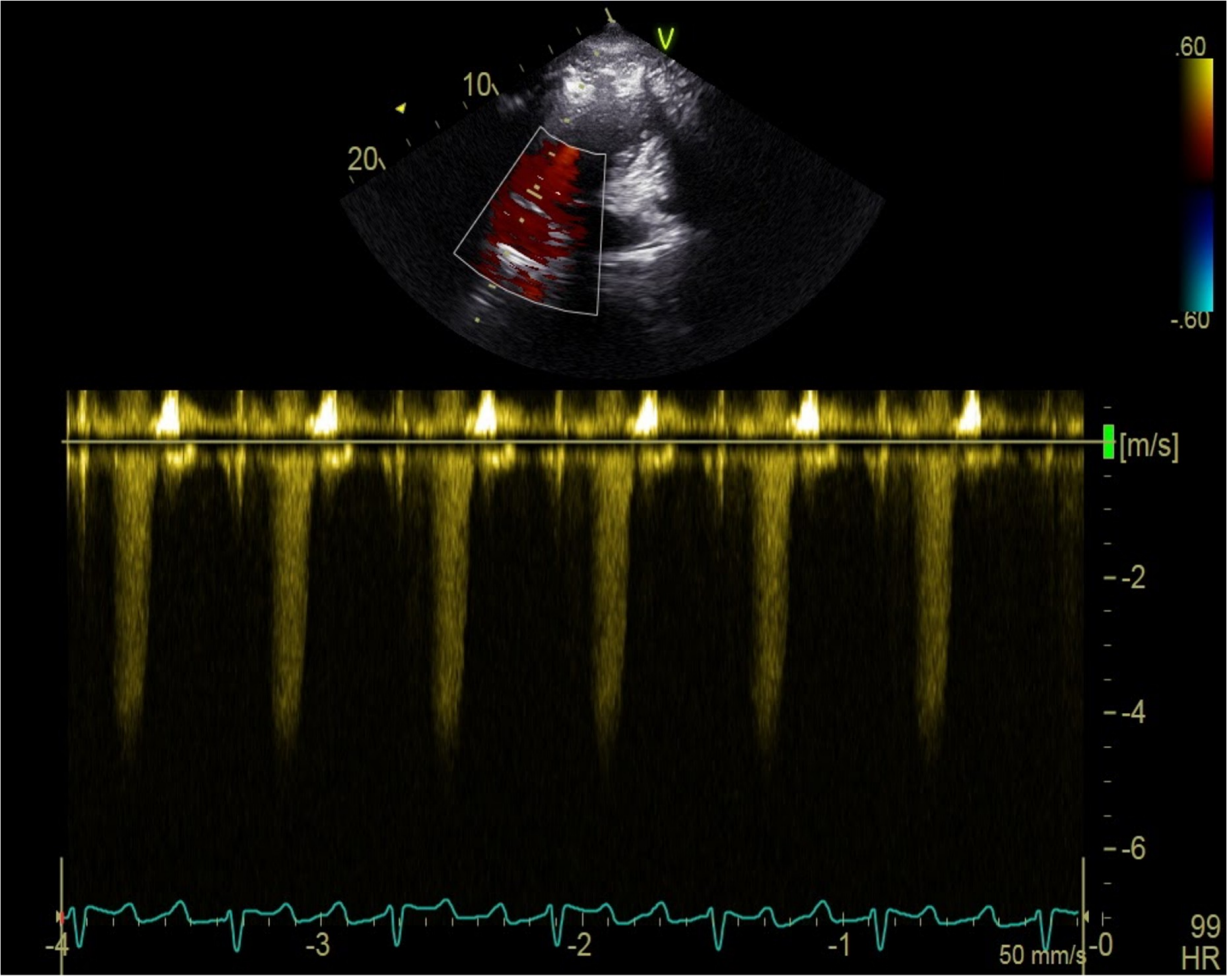
Pulmonary hypertension in a BB dolphin. Continuous wave Doppler measurement of tricuspid regurgitation in an adult BB dolphin diagnosed with pulmonary hypertension.

Additionally, abnormalities associated with the pulmonary artery were observed in BB. Two BB dolphins (one subadult male, estimated to be between 5.0–6.5 years old [50], and one adult female) were diagnosed with pulmonary hypertension based on Doppler-derived pulmonary artery pressures from tricuspid regurgitation (Fig 4). One of these dolphins had a dilated main pulmonary artery, while the other did not have adequate pulmonary artery visualization to discern. The maximum pulmonic regurgitant flow velocity was used to estimate pulmonary arterial pressure using the modified Bernoulli equation, and both dolphins had estimated pulmonary arterial pressure well above the established cutoffs in other species [39] for diagnosing pulmonary hypertension. One dolphin had moderately elevated pulmonary arterial pressure, 66mmHg, and the second dolphin had severely elevated pulmonary arterial pressure, 91mmHg. Standard categories in domestic animals were utilized, with mild pulmonary hypertension defined as pulmonary arterial pressure 30-50mmHg, moderate 51-75mmHg, and severe > 75mmHg [39].

### Electrocardiograms

ECGs were recorded on 37 dolphins (16 SB, 21 BB) opportunistically, totaling over 1,086 minutes of ECG recordings. In SB, 12 dolphins had ECGs recorded in the water and 12 while on the boat deck (8 dolphins recorded both in the water and on the boat deck). In BB, 21 dolphins were recorded in the water and two on the boat deck (two animals were recorded both in the water and on the boat deck). The quality of the ECG tracings was generally better when the animal was on the padded boat deck versus in the water, as there were more frequent motion artifacts due to movement of the water, the handlers, the dolphin itself, or a combination thereof.

**Fig 5:**
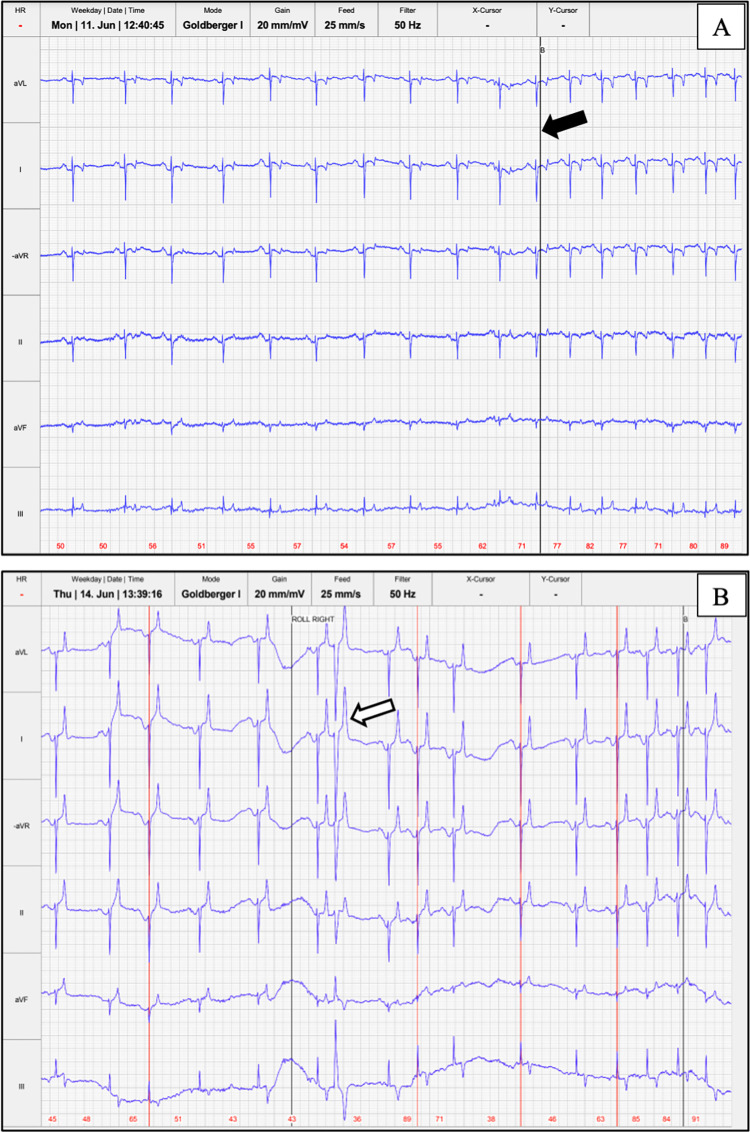
Telemetric ECG recordings from two SB dolphins. (A) Demonstrating the normal, pronounced respiratory sinus arrhythmia, with timing of the breath marked with the black line (black arrow). (B) Demonstrating respiratory sinus arrhythmia with an isolated ventricular premature complex (white arrow). Lead I is modified to the base-apex lead, and the primarily negative depolarization in base-apex and Lead II is normal for this type B cardiac conduction system. The paper speed is 25mm/s and gain is 20mm/mV, with the sweep covering 16 seconds.

Various arrhythmias were detected in 43% of dolphins in BB (9 of 21) and 31% of dolphins in SB (5 of 16), with no significant difference between site (*p* = 0.35) or sex by site (males *p* = 0.78, females *p* = 0.15). All dolphins had a normal respiratory sinus arrhythmia. The other arrhythmias detected included, in BB: isolated premature complexes (atrial premature complexes, ventricular premature complexes, and junctional premature complexes), period of sinus bradycardia, and second degree atrioventricular block (Mobitz Type 2); and in SB: isolated premature complexes (atrial premature complexes, ventricular premature complexes), periods of sinus tachycardia, and possible ST segment elevation. All of these arrhythmias were considered to be low to moderate risk for a cardiac event. Fig 5 depicts an example of ECG recording with respiratory sinus arrhythmia and a recording with an isolated premature complex.

None of the arrhythmias found on ECG were heard on auscultation, nor on the brief ECG performed during the echocardiograms. There were no significant differences between the lowest and highest heart rates by site (*p* = 0.1), nor change in heart rate (split) by site (*p* = 1).

### Murmur prevalence

Linnehan *et al*. [1] previously described murmur prevalence and characteristics for SB and BB dolphins, reporting that 17 of 19 dolphins ausculted in SB had systolic murmurs (89%) and 30 of 34 dolphins ausculted in BB had systolic murmurs (88%). Spectral Doppler was used to measure flow velocities across the outflow tracts, and almost all (95%, 38/40) dolphins with audible murmurs had peak outflow velocities ≥1.6m/s. Three dolphins also had medium valvular regurgitation which could be the source of their murmurs. The presence of audible murmurs in most of the free-ranging dolphins (88%) was attributed to high velocity blood flow in the absence cardiac disease based on echocardiography (*i*.*e*., innocent flow murmurs) [1].

### Histopathology

**Fig 6:**
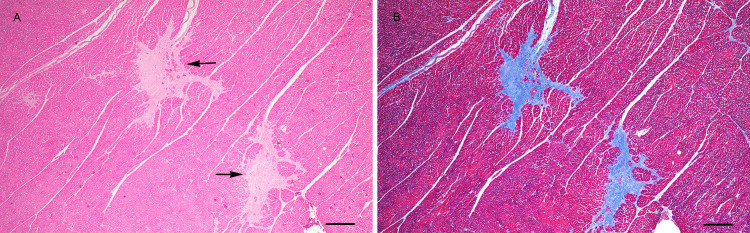
Photomicrograph of the heart from a bottlenose dolphin that stranded in 2012 in Alabama with severe fibrosis. A. Hematoxylin and eosin stained section of left ventricle. Note the pale staining fibrous connective tissue replacing and surrounding myocytes (arrows). B. Masson’s trichrome stained section of the same region of the left ventricle with severe fibrosis. Bar = 200 μm.

In the dolphins who stranded and received postmortem examinations, prevalence of myocardial fibrosis was significantly higher in NGoM dolphins compared to reference dolphins (46% NGoM vs 19% reference, *p* = 0.003) (Table 4). In affected tissue, there were focal to multifocal areas in which sparsely cellular fibrous connective tissue replaced and/or surrounded myocytes (Fig 6). Occasional entrapped myofibers encircled by fibrous connective tissue were shrunken, had enlarged karyomegalic nuclei and/or contained small to moderate amounts of lipofuscin. The ventricles and interventricular septum were most commonly affected, however, there was no observed difference in fibrosis distribution between dolphins in the NGoM and control group. Fibrosis was graded as mild (*n* = 30 NGoM; *n* = 6 reference), moderate (*n* = 12 NGoM; *n* = 2 reference), or severe (*n* = 5 NGoM; *n* = 0 reference) however there was no significant difference among severity score prevalence between groups. Dolphins with myocardial fibrosis in the NGoM group (age range: 5–34 years; age average: 19.8 years; length range: 195–290 cm; length average: 242.3 cm) were significantly older (*p*<0.0001) and longer (*p*<0.0001) than dolphins without fibrosis (age range: 2–29 years; age average: 9.0 years; length range 117–263 cm, length average 196.8 cm). Reference dolphins with fibrosis (age range: 13–50; age average: 28; length range: 190–274 cm; length average 232.4 cm) were also significantly older (*p* = 0.02) than those without fibrosis (age range: 3 months– 44 years; age average 12 years; length range: 122–278 cm; length average 211.1 cm). There was no significant difference in age or length between the NGoM and reference populations, indicating that the difference in fibrosis was not likely driven by differences in age between the groups. Lesions consistent with heart failure were observed in six dolphins stranded in the NGoM, all of which had interstitial fibrosis as a consistent lesion. Of the six dolphins with heart failure, five had moderate to severe interstitial fibrosis. One dolphin diagnosed with heart failure had significant cardiac abnormalities noted on gross examination including pericardial effusion, a flaccid and thin right ventricular wall, pale streaking along the epicardial surface and myocardial cut section, and moderate nodular thickening of the mitral and tricuspid valves). On cut section the ventricular myocardium had pale tan streaks corresponding to the areas of fibrosis noted histologically (S1 Fig and Fig 6). No dolphins either with or without myocardial fibrosis had plexiform vascular lesions suggestive of pulmonary hypertension. Additional lung lesions identified in both NGoM and reference dolphins included bacterial pneumonia, angiomatosis, and lesions (granulomas, pneumonia and fibrosis) consistent with lungworm-related pneumonia as previously described and compared between groups [16].

**Table 4:** Prevalence of heart lesions identified histopathologically in dolphins stranding in the NGoM from 2010-February 2019 compared to the lesion prevalence in reference dolphins.

Histopathology parameter	Reference cases	NGoM cases	Reference prevalence (95% CI)	NGoM prevalence (95% CI)	Relative risk	*p-*value
Arteriosclerosis	2 / 42	8 / 100	0.05 (0–0.17)	0.08 (0.04–0.15)	1.68	0.723
Degeneration and/or necrosis	2 / 42	16 / 100	0.05 (0–0.17)	0.16 (0.1–0.25)	3.36	0.096
Heart failure	0 / 42	6 / 100	0 (0–0.1)	0.06 (0.03–0.13)	NA	0.179
Lipofuscinosis	13 / 42	38 / 100	0.31 (0.19–0.46)	0.38 (0.29–0.48)	1.23	0.451
Myocardial fibrosis	8 / 42	46 / 100	0.19 (0.1–0.34)	0.46 (0.37–0.56)	2.42	**0.003**
Myocardial karyomegaly	16 / 42	50 / 100	0.38 (0.25–0.53)	0.5 (0.4–0.6)	1.31	0.204
Myocardial nuclear rowing	28 / 42	70 / 100	0.67 (0.51–0.79)	0.7 (0.6–0.78)	1.05	0.696
Myocarditis	3 / 42	14 / 100	0.07 (0.02–0.2)	0.14 (0.08–0.22)	1.96	0.396

*P*-values ≤ 0.05 are reported in bold.

Further details regarding histopathology results are provided in the publicly available data set through Gulf of Mexico Research Initiative Information & Data Cooperative (GRIIDC) at https://data.gulfresearchinitiative.org/data/R6.x809.000:0002.

## Discussion

This prospective study is the first to comprehensively examine the cardiac health of free-ranging dolphins and to examine the potential chronic cardiotoxic effects of oil exposure on cetaceans. Dolphins breathe at the air-water interface, where high concentrations of cardiotoxic volatile compounds can be found, and may be quickly absorbed into the bloodstream [2, 6]. In addition to direct cardiotoxic effects, chronic pulmonary disease represents another potential pathway for cardiac injury. A high prevalence of pulmonary disease was observed in dolphins within the oil spill footprint [19], which may continue as a chronic disease state in the surviving dolphins [15]. Heart disease is a common sequela to chronic lung dysfunction in humans and can cause increased vascular resistance, pulmonary hypertension, and resulting cardiac hypertrophy, dilation, or failure [52]. Therefore, dolphins surviving the DWH disaster but sustaining pulmonary injury and developing chronic pulmonary disease could be at an increased risk of secondary cardiac disease.

There were several significant echocardiographic abnormalities identified in BB dolphins in this study. The most clinically relevant finding was the diagnosis of pulmonary hypertension in two BB dolphins, which has not been previously reported in wild dolphins. To the authors’ knowledge, the only previous report of pulmonary hypertension in a live cetacean is from a 1968 study, in which a managed dolphin had severe pulmonary hypertension (mean pulmonary arterial pressure 122 mmHg measured via direct right heart catheterization) due to severe lungworm infestation obstructing the pulmonary artery [53]. The gold standard for diagnosis of pulmonary hypertension is by direct catheterization of the right heart [37, 54]. Given the logistical challenges associated with invasive, direct pulmonary arterial catheterization, techniques for non-invasively estimating the pulmonary arterial systolic pressure using echocardiography are well-established and widely used in human and domestic animal medicine, which typically generates comparable measurements with more direct techniques [37–40]. In the current study, pulmonary artery pressures were estimated following these non-invasive methods using Doppler echocardiography. Both dolphins diagnosed with hypertension had high tricuspid regurgitation velocity and estimated pressures well above the normal reference range, with one dolphin in the moderate category (66 mmHg) and the other severe (91 mmHg), extrapolating from human and domestic animal reported ranges [39, 54]. In addition to elevated pressures, other supporting echocardiographic findings to diagnose pulmonary hypertension include right heart enlargement and/or dilated pulmonary arteries [54]. One of the BB dolphins with pulmonary hypertension had a dilated main pulmonary artery and mild right atrial dilation; the other dolphin with pulmonary hypertension had inadequate visualization of the pulmonary artery to discern any potential dilation.

Pulmonary arterial hypertension is a well-known complication of numerous cardiorespiratory and systemic diseases, and is a common sequela to chronic pulmonary disease. In the two dolphins with pulmonary hypertension in BB, there are two potential pathways based on our current knowledge of the population and individual health assessments: 1) direct cardiotoxic effects of oil causing primary pulmonary arterial hypertension, and/or 2) chronic lung disease. One of these dolphins was an adult female alive at the time of the oil spill, making both pathways are plausible in her case, while the second dolphin was likely born 1.5 to 3 years post-spill, and therefore was not exposed to oil during the spill itself, but could have been exposed to oil while *in utero* or from sediment ingestion after birth, as oil residue persisted in the region for several years [55–57]. Both dolphins had evidence of pulmonary disease on ultrasound, supporting the chronic lung disease pathway. Lungworm infestation, like that reported in a managed dolphin with pulmonary hypertension [53], cannot be definitively ruled out as the etiology; however, severe lungworm-related changes were not identified with cardiac or thoracic ultrasound in either of the dolphins in the current study, and it is therefore considered less likely. While the exact etiology of pulmonary hypertension cannot be definitively determined without further diagnostics, based on the other examination findings and population health, we hypothesize that the adult dolphin developed chronic pulmonary disease related to oil exposure, subsequent increased vascular resistance, and development of pulmonary hypertension. Interestingly, both dolphins with pulmonary hypertension were noted to have abnormal thoracic auscultation prior to their echocardiograms, with the adult dolphin having crackles and the younger dolphin having harsh bronchovesicular sounds. The only other dolphin in the 2018 health assessments reported to have harsh bronchovesicular sounds did not receive an echocardiogram. This highlights the importance of cardiothoracic auscultation and suggests that dolphins with harsh lung sounds or crackles warrant further diagnostics to rule out cardiopulmonary disease, including pulmonary hypertension. None of the histologically evaluated dolphins, including animals with myocardial fibrosis, had overt lesions such as intimal remodeling or plexiform vascular change described in association with pulmonary hypertension in other species. Histologic lesions due pulmonary hypertension have not been described in cetaceans. As many dolphins did have lung lesions due to various causes, future histologic studies on free-ranging dolphins should attempt to better characterize both primary and secondary pulmonary vascular lesions. Additionally, future studies to define normal pulmonary artery pressures in dolphins would be beneficial for clinicians to better diagnose and categorize pulmonary hypertension in this species.

Another notable difference between the two free-ranging populations was that BB dolphin hearts had significantly thinner left ventricular walls than SB dolphin hearts, as measured by IVSd and LVPWd. It is unclear if this difference in ventricular thickness is related to physiologic differences between the two populations, or to cardiotoxic effects of oil, or due to other unrelated disease processes. Considering physiologic or athletic differences between the two populations, diving behavior, home ranges, and environments must be assessed. Large variations in dive behavior and home range exist within bottlenose dolphins [58–60]. Differences in cardiac output between the deep-diving and estuarine/coastal dolphins have been documented and hypothesized to be a result of their different diving behavior [61]. Estuarine/coastal bottlenose dolphins, like those in SB and BB, live close to shore and tend to perform short, shallow dives [58], versus offshore dolphins, living in deeper waters and routinely deep diving [62]. SB dolphins are active throughout day and night and spend up to 20% of the day foraging [63]. Studies of the estuarine BB dolphins have described a small home range and high site fidelity, similar to SB dolphins [64]. There is no published information, however, regarding the diving behavior of BB dolphins. BB dolphins may not have to dive deep very often, as most of the surrounding waters are shallow estuarine habitat, however there are deeper waters in the passes between the barrier islands and in the open Gulf waters. These behavioral differences could account for overall differences in athleticism between the two groups and manifest as changes to their hearts over time.

In human athletes, exercise-induced cardiac remodeling is well-studied [65–68]. Different types of exercise can also affect the degree and character of the adaptations due to different relative amounts of isotonic and isometric stress on the heart [65, 67, 68]. For example, distance runners have significantly different cardiac adaptations than rowers [65], and elite swimmers have demonstrable cardiac differences from runners [68]. Given the variability between heart indices across humans with varying exercise, it is plausible that dolphins can have differences in heart indices due to differences in exercise as it relates to their diving, ranging, and foraging behaviors.

Another possibility is that the secondary effects of oil exposure have caused a de-conditioning of the heart muscle in BB dolphins, in an opposite transition from that described in athletes, resulting in thinner heart walls. Further work is needed to study additional managed and free-ranging dolphins with known activity levels to adequately investigate this theory.

The other possible explanation for thin left ventricular walls in BB dolphins is due to direct cardiotoxic effects of oil, as documented in other species. Oil exposure could lead to thinning ventricular walls as a result of decreased systolic function, infarction, necrosis, fibrosis, and possibly other mechanisms. In double-crested cormorants with dermal exposure to MC252 oil, hearts at necropsy had softer cardiac musculature, described grossly as soft, flaccid, and enlarged, and some birds had cardiac fibrosis on histopathology [9]. The birds had decreased fractional shortening, indicating decreased systolic function. In sheep exposed to crude oil by ingesting contaminated water in an extraction well, gross necropsy revealed flaccid hearts with edema and coagulative necrosis of myofibers on histopathology [69]. Cattle exposed via inhalation and possible ingestion to sour multiphase crude petroleum also had enlarged and “flabby” hearts upon gross examination [70]. These cardiac abnormalities in other oil-impacted species are similar to the cardiac abnormalities documented in BB dolphins. Continued echocardiographic studies and histopathological evaluation of cardiac tissue on BB dolphins are needed to further investigate oil exposure as a possible etiology for the thin ventricular hearts in BB dolphins.

The histopathologic findings from NGoM dolphins available for examination in this study showed that dolphins stranding in the NGoM oil spill footprint had a significantly higher prevalence of myocardial fibrosis compared to dolphins outside of the oil spill footprint, regardless of age. One of the dolphins with heart failure had flaccid, thin ventricular walls, similar to the oil-impacted hearts described in other species. A number of cardiac insults can lead to myocardial fibrosis including toxic insult, bacterial, viral, or protozoal infection, biotoxicosis, and regional hypoxia. In the affected dolphins, it was not possible to determine the exact cause of fibrosis. Previous biotoxin analysis completed on stranded dolphins during large portions of this study period did not identify significant toxin levels [16]. While the etiology of cardiac fibrosis cannot be determined, the findings support oil cardiotoxicity as a possibility. It should be noted that the stranded dolphins in this study were not a random sample of the stranded dolphin population, and the true prevalence of cardiac fibrosis in free ranging dolphins is likely lower than noted in this study. Continued necropsy and histopathology work will be highly valuable to further elucidate the mechanisms for cardiac changes in dolphins that are related to oil exposure and/or other stressors. Further, dolphin necropsy protocols should include thorough evaluation of cardiac wall thickness and more standardized sampling protocols for heart tissue so that pathogenesis of heart disease in dolphins can be better evaluated.

Telemetric ECG was beneficial in this study for patient monitoring during health assessments and for identification of arrhythmias. Arrhythmias were detected at a similar prevalence in BB and SB (in 43% and 31% of dolphins, respectively) regardless of location. All dolphins had a respiratory sinus arrhythmia, which is normal for this species [24, 26], and the most commonly identified arrhythmias were isolated premature complexes and isolated instances of second degree atrioventricular block (Fig 5). No life-threatening arrhythmias were seen. In horses, similar isolated arrhythmias are seen commonly due to high vagal tone and are not necessarily indicative of cardiac pathology, especially if they are infrequent, isolated, and disappear with exercise [43, 71–75]. In dolphins, isolated premature complexes have been reported in dolphins at rest [25], as well as during dives, and are thought to be related to the interplay of sympathetic and parasympathetic tone [26], as in horses. In the authors’ experience, isolated premature complexes or isolated instances of atrioventricular block are seen fairly commonly in managed dolphins both in and out of water, and are of little clinical significance, though true prevalence studies have yet to be performed. Pathologic arrhythmias can occur in dolphins, including ventricular tachycardia and sustained high grade second degree atrioventricular block reported in managed dolphins [76]. Telemetric ECG is useful in identifying arrhythmias that may be transient and can be easily missed with auscultation or a brief ECG, as demonstrated in this study.

Prior to this study, no baseline data on the cardiac health of free-ranging dolphins existed, and there were limited data published regarding normal managed dolphin cardiac parameters. Studying Navy dolphins for echocardiogram technique refinement highlighted some interesting differences between the managed and free-ranging dolphins. Managed Navy dolphins had more challenging echocardiogram windows, with lung interference causing a smaller window where the heart could be visualized. One hypothesis to explain the more challenging visualization in the Navy dolphins could be the differences in prevalence of pulmonary disease, with the Navy population having a lower prevalence of pulmonary disease than the free-ranging dolphins, resulting in more air-filled lung tissue and subsequent air artifact. Additional cardiac exams are needed on managed and free-ranging dolphins to confidently determine what is normal for a given population with unique activity levels and health status.

The long period of time between the oil spill and the first assessments of cardiac health on the surviving dolphin population in BB was not ideal. Many of the studies in other species have examined the acute cardiotoxic effects of oil in controlled laboratory exposures. There are few long-term or follow-up studies of surviving animals to examine how the heart may adapt and change over time after oil exposure. In Zheng *et al*, rats were exposed to oil for a short period of time (6 hours) and the acute cardiac changes observed at one day post-exposure resolved at 28 days [8]. Given the magnitude and severity of the DWH oil spill, the period of oil exposure was likely much longer, and if the acute effects were reversible, it is unknown how long that would take. In BB dolphins, it is possible that those surviving the initial injuries were able to adapt, and their hearts have since adapted to sublethal, direct cardiotoxic effects. In long-term studies on the human oil spill workers and residents in close proximity, cardiac effects have been demonstrated 5 to 7 years after the spill, with increased risk of myocardial infarction [12, 13]. Since we were not able to examine the cardiac health of the dolphins prior to the spill, immediately after the spill, nor at other time points prior to our study 8 years later, we cannot determine how the cardiac health has possibly changed over time. This challenge illustrates the importance of studying baseline physiology and health of animal populations, via live health assessments and necropsy examinations, both before and after environmental disasters.

## Conclusion

Oil-impacted dolphins in BB showed evidence of cardiac abnormalities, including significantly thinner left ventricular walls, smaller left atria, and a higher prevalence of valvular abnormalities. Additionally, two dolphins in BB were diagnosed with pulmonary hypertension, which has not been previously reported in wild dolphins. The cardiac abnormalities identified in BB dolphins are similar to those seen in other species with documented oil cardiotoxicity, however, future work is needed to rule out other hypotheses and further elucidate the connection between oil exposure, pulmonary disease, and the observed cardiac abnormalities. Continued live and deceased animal studies on free-ranging dolphins and those under managed care are necessary to advance our understanding of cardiac disease and to further investigate the observed differences among populations. This prospective study has successfully expanded our knowledge and capability regarding dolphin cardiac health.

## Supporting information

S1 Fig Heart from a wild bottlenose dolphin that stranded in Alabama in 2019 and was diagnosed with heart failure. (A) Note the thin right ventricle and pale streaking throughout the epicardium. (B) There is pale tan streaking of the left ventricular endocardial surface and nodular thickening of the leaflets of the mitral valve (endocardiosis).
(TIF)

## References

[1] Linnehan BK, Hsu A, Gomez FM, Huston SM, Takeshita R, Colegrove KM, et al. Standardization of dolphin cardiac auscultation and characterization of heart murmurs in managed and free-ranging bottlenose dolphins (*Tursiops truncatus*). Front Vet Sci. 2020;7. Available from: https://www.frontiersin.org/articles/10.3389/fvets.2020.570055/full 33240948 10.3389/fvets.2020.570055 PMC7678442

[2] Brette F, Machado B, Cros C, Incardona JP, Scholz NL, Block BA. Crude oil impairs cardiac excitation-contraction coupling in fish. Science. 2014 Feb 14;343(6172):772–6. doi: 10.1126/science.1242747 24531969

[3] Incardona JP, Gardner LD, Linbo TL, Brown TL, Esbaugh AJ, Mager EM, et al. Deepwater Horizon crude oil impacts the developing hearts of large predatory pelagic fish. PNAS. 2014 Apr 15;111(15):E1510–8. doi: 10.1073/pnas.1320950111 24706825 PMC3992643

[4] Incardona JP. Molecular mechanisms of crude oil developmental toxicity in fish. Arch Environ Contam Toxicol. 2017 Jul 1;73(1):19–32. doi: 10.1007/s00244-017-0381-1 28695261

[5] Nelson D, Heuer RM, Cox GK, Stieglitz JD, Hoenig R, Mager EM, et al. Effects of crude oil on *in situ* cardiac function in young adult mahi-mahi (*Coryphaena hippurus*). Aquat Toxicol. 2016 Nov;180:274–81. doi: 10.1016/j.aquatox.2016.10.012 27768947

[6] Brette F, Shiels HA, Galli GLJ, Cros C, Incardona JP, Scholz NL, et al. A novel cardiotoxic mechanism for a pervasive global pollutant. Sci Rep. 2017 Jan 31;7:41476. doi: 10.1038/srep41476 28139666 PMC5282528

[7] Perrichon P, Mager EM, Pasparakis C, Stieglitz JD, Benetti DD, Grosell M, et al. Combined effects of elevated temperature and Deepwater Horizon oil exposure on the cardiac performance of larval mahi-mahi, Coryphaena hippurus. PLOS ONE. 2018 Oct 17;13(10):e0203949. doi: 10.1371/journal.pone.0203949 30332409 PMC6192557

[8] Zheng W, McKinney W, Kashon M, Jackson M, Law B, Kan H, et al. The effects of crude oil vapor exposure on the cardiovascular system of rats. In: Toxicologist. Baltimore, Maryland; 2017. p. 479.

[9] Harr KE, Rishniw M, Rupp TL, Cacela D, Dean KM, Dorr BS, et al. Dermal exposure to weathered MC252 crude oil results in echocardiographically identifiable systolic myocardial dysfunction in double-crested cormorants (*Phalacrocorax auritus*). Ecotoxicol Environ Saf. 2017 Dec;146:76–82. doi: 10.1016/j.ecoenv.2017.04.010 28666537

[10] Strelitz J, Engel LS, Kwok RK, Miller AK, Blair A, Sandler DP. *Deepwater Horizon* oil spill exposures and nonfatal myocardial infarction in the GuLF STUDY. Environ Health. 2018 Aug 25 [cited 2019 Aug 5];17. Available from: https://www.ncbi.nlm.nih.gov/pmc/articles/PMC6109340/ doi: 10.1186/s12940-018-0408-8 30144816 PMC6109340

[11] Strelitz J, Engel L, Kwok R, Miller A, Blair A, Sandler D. O29-3 Oil spill cleanup work and incident coronary heart disease in the gulf study. Occup Environ Med. 2016 Sep 1;73(Suppl 1):A54–A54.

[12] Strelitz J, Sandler DP, Keil AP, Richardson DB, Heiss G, Gammon MD, et al. Exposure to total hydrocarbons during cleanup of the *Deepwater Horizon* oil spill and risk of heart attack across 5 years of follow-up. Am J Epidemiol. 2019 May 1;188(5):917–27. doi: 10.1093/aje/kwz017 30698634 PMC6494668

[13] Strelitz J, Keil AP, Richardson DB, Heiss G, Gammon MD, Kwok RK, et al. Self-reported myocardial infarction and fatal coronary heart disease among oil spill workers and community members 5 years after *Deepwater Horizon*. Environ Res. 2019;168:70–9. doi: 10.1016/j.envres.2018.09.026 30278364 PMC6263782

[14] D’Andrea MA, Reddy GK. The development of long-term adverse health effects in oil spill cleanup workers of the *Deepwater Horizon* offshore drilling rig disaster. Front Public Health. 2018 [cited 2019 Aug 5];6. Available from: https://www.frontiersin.org/articles/10.3389/fpubh.2018.00117/full#B33 29755965 10.3389/fpubh.2018.00117 PMC5932154

[15] Smith CR, Rowles TK, Hart LB, Townsend FI, Wells RS, Zolman ES, et al. Slow recovery of Barataria Bay dolphin health following the *Deepwater Horizon* oil spill (2013–2014), with evidence of persistent lung disease and impaired stress response. Endanger Species Res. 2017 Jan 31;33:127–42.

[16] Venn-Watson S, Colegrove KM, Litz J, Kinsel M, Terio K, Saliki J, et al. Adrenal gland and lung lesions in Gulf of Mexico common bottlenose dolphins (*Tursiops truncatus*) found dead following the *Deepwater Horizon* oil spill. PLOS ONE. 2015 May 20;10(5):e0126538. doi: 10.1371/journal.pone.0126538 25992681 PMC4439104

[17] Venn-Watson S, Garrison L, Litz J, Fougeres E, Mase B, Rappucci G, et al. Demographic clusters identified within the Northern Gulf of Mexico common bottlenose dolphin (*Tursiops truncatus*) unusual mortality event: January 2010—June 2013. PLOS ONE. 2015 Feb 11;10(2):e0117248. doi: 10.1371/journal.pone.0117248 25671657 PMC4324990

[18] Lane Suzanne M., Smith Cynthia R., Mitchell Jason, Balmer Brian C., Barry Kevin P., McDonald Trent, et al. Reproductive outcome and survival of common bottlenose dolphins sampled in Barataria Bay, Louisiana, USA, following the *Deepwater Horizon* oil spill. Proc Royal Soc B. 2015 Nov 7;282(1818):20151944. doi: 10.1098/rspb.2015.1944 26538595 PMC4650159

[19] Schwacke LH, Smith CR, Townsend FI, Wells RS, Hart LB, Balmer BC, et al. Health of common bottlenose dolphins (*Tursiops truncatus*) in Barataria Bay, Louisiana, following the *Deepwater Horizon* oil spill. Environ Sci Technol. 2014 Jan 7;48(1):93–103. doi: 10.1021/es403610f 24350796

[20] Schwacke LH, Thomas L, Wells RS, McFee WE, Hohn AA, Mullin KD, et al. Quantifying injury to common bottlenose dolphins from the *Deepwater Horizon* oil spill using an age-, sex- and class-structured population model. Endanger Species Res. 2017 Jan 31;33:265–79.

[21] Takeshita R, Sullivan L, Smith C, Collier T, Hall A, Brosnan T, et al. The *Deepwater Horizon* oil spill marine mammal injury assessment. Endanger Species Res. 2017 Jan 31;33.

[22] De Guise S, Levin M, Cote E, Jasperse L, Hart B, Smith C, et al. Changes in immune functions in bottlenose dolphins in the northern Gulf of Mexico associated with the *Deepwater Horizon* oil spill. Endanger Species Rese. 2017 May 18;33.

[23] Colegrove KM, Venn-Watson S, Litz J, Kinsel MJ, Terio KA, Fougeres E, et al. Fetal distress and in utero pneumonia in perinatal dolphins during the Northern Gulf of Mexico unusual mortality event. Dis Aquat Organ. 2016 Apr 12;119(1):1–16. doi: 10.3354/dao02969 27068499

[24] Harms CA, Jensen ED, Townsend FI, Hansen LJ, Schwacke LH, Rowles TK. Electrocardiograms of bottlenose dolphins (*Tursiops truncatus*) out of water: habituated collection versus wild postcapture animals. J Zoo Wildl Med. 2013 Dec;44(4):972–81. doi: 10.1638/2013-0093.1 24450057

[25] Yaw TJ, Kraus MS, Ginsburg A, Clayton LA, Hadfield CA, Gelzer AR. Comparison of a smartphone-based electrocardiogram device with a standard six-lead electrocardiogram in the Atlantic bottlenose dolphin (*Tursiops truncatus*). J Zoo Wildl Med. 2018 Sep;49(3):689–95. doi: 10.1638/2017-0140.1 30212343

[26] Williams TM, Fuiman LA, Kendall T, Berry P, Richter B, Noren SR, et al. Exercise at depth alters bradycardia and incidence of cardiac anomalies in deep-diving marine mammals. Nat Commun. 2015 Jan 16;6:6055. doi: 10.1038/ncomms7055 25592286

[27] Bickett N, Tift M, St. Leger J, Ponganis P. Heart rate regulation in the killer whale. FASEB J. 2016 Apr 1;30 (1_supplement):1230.9–1230.9.

[28] Elmegaard SL, Johnson M, Madsen PT, McDonald BI. Cognitive control of heart rate in diving harbor porpoises. Curr Biol. 2016 Nov 21;26(22):R1175–6. doi: 10.1016/j.cub.2016.10.020 27875692

[29] Goldbogen JA, Cade DE, Calambokidis J, Czapanskiy MF, Fahlbusch J, Friedlaender AS, et al. Extreme bradycardia and tachycardia in the world’s largest animal. PNAS. 2019 Dec 10;116(50):25329–32. doi: 10.1073/pnas.1914273116 31767746 PMC6911174

[30] Sklansky M, Levine G, Havlis D, West N, Renner M, Rimmerman C, et al. Echocardiographic evaluation of the bottlenose dolphins (*Tursiops truncatus*). J Zoo Wildl Med. 2006 Dec;37(4):454–63. doi: 10.1638/05-116.1 17315429

[31] Miedler S, McBain J, Reidarson T, Schmitt T. Handbook of transthoracic cardiac ultrasound examination in bottlenose dolphins. Vienna; 2008.

[32] Miedler S, Fahlman A, Valls Torres M, Álvaro Álvarez T, Garcia-Parraga D. Evaluating cardiac physiology through echocardiography in bottlenose dolphins: using stroke volume and cardiac output to estimate systolic left ventricular function during rest and following exercise. J Exp Biol. 2015 Nov;218(Pt 22):3604–10. doi: 10.1242/jeb.131532 26385334

[33] Chetboul V, Lichtenberger J, Mellin M, Mercera B, Hoffmann A-C, Chaix G, et al. Within-day and between-day variability of transthoracic anatomic M-mode echocardiography in the awake bottlenose dolphin (*Tursiops truncatus*). J Vet Cardiol. 2012 Dec;14(4):511–8. doi: 10.1016/j.jvc.2012.07.002 23102806

[34] Barratclough A, Wells RS, Schwacke LH, Rowles TK, Gomez FM, Fauquier DA, et al. Health assessments of common bottlenose dolphins (*Tursiops truncatus*): past, present, and potential conservation applications. Front Vet Sci. 2019 [cited 2020 May 19];6. Available from: https://www.frontiersin.org/articles/10.3389/fvets.2019.00444/full 10.3389/fvets.2019.00444 PMC6923228 31921905

[35] Wells RS. Social Structure and Life History of Bottlenose Dolphins Near Sarasota Bay, Florida: Insights from Four Decades and Five Generations. In: Yamagiwa J, Karczmarski L, editors. Primates and Cetaceans: Field Research and Conservation of Complex Mammalian Societies. Tokyo: Springer Japan; 2014 [cited 2019 Sep 17]. p. 149–72. (Primatology Monographs). Available from: 10.1007/978-4-431-54523-1_8

[36] Otto CM. Textbook of Clinical Echocardiography E-Book. Elsevier Health Sciences; 2013. 572 p.

[37] Serres F, Chetboul V, Gouni V, Tissier R, Sampedrano CC, Pouchelon J-L. Diagnostic value of echo-doppler and tissue doppler imaging in dogs with pulmonary arterial hypertension. J Vet Int Med. 2007;21(6):1280–9. doi: 10.1892/07-064.1 18196738

[38] Stephen B, Dalal P, Berger M, Schweitzer P, Hecht S. Noninvasive estimation of pulmonary artery diastolic pressure in patients with tricuspid regurgitation by Doppler echocardiography. Chest. 1999 Jul;116(1):73–7. doi: 10.1378/chest.116.1.73 10424506

[39] Serres FJ, Chetboul V, Tissier R, Carlos Sampedrano C, Gouni V, Nicolle AP, et al. Doppler echocardiography–derived evidence of pulmonary arterial hypertension in dogs with degenerative mitral valve disease: 86 cases (2001–2005). JAVMA. 2006 Dec 1;229(11):1772–8. doi: 10.2460/javma.229.11.1772 17144824

[40] Uehara Y. An attempt to estimate the pulmonary artery pressure in dogs by means of pulsed Doppler echocardiography. J Vet Med Sci. 1993 Apr;55(2):307–12. doi: 10.1292/jvms.55.307 8513015

[41] Kitabatake A, Inoue M, Asao M, Masuyama T, Tanouchi J, Morita T, et al. Noninvasive evaluation of pulmonary hypertension by a pulsed Doppler technique. Circulation. 1983 Aug;68(2):302–9. doi: 10.1161/01.cir.68.2.302 6861308

[42] Vasan Ramachandran S., Larson Martin G., Levy Daniel. Determinants of echocardiographic aortic root size. Circulation. 1995 Feb 1;91(3):734–40. doi: 10.1161/01.cir.91.3.734 7828301

[43] Patteson M. Equine Cardiology. Wiley; 1996. 264 p.

[44] Hamlin RL, Jackson RF, Himes JA, Pipers FS, Townsend AC. Electrocardiogram of bottle-nosed dolphin (*Tursiops truncatus*). Am J Vet Res. 1970 Mar;31(3):501–5. 5443944

[45] Levine SA. The systolic murmur: its clinical significance. JAMA. 1933 Aug 5;101(6):436–8.

[46] Zipes DP, Libby P, Bonow RO, Mann DL, Tomaselli GF. Braunwald’s Heart Disease E-Book: A Textbook of Cardiovascular Medicine. Elsevier Health Sciences; 2018. 2142 p.

[47] Tilley LP. Manual of Canine and Feline Cardiology. Elsevier Health Sciences; 2008. 464 p.

[48] Mcfee W. Age distribution and growth of two bottlenose dolphin (*Tursiops truncatus*) populations from capture-release studies in the Southeastern United States. Aquat Mamm. 2012 Mar 1;38:17–30.

[49] Hohn AA, Scott MD, Wells RS, Sweeney JC, Irvine AB. Growth layers in teeth from known-age, free-ranging bottlenose dolphins. Mar Mamm Sci 1989;5(4):315–42.

[50] Herrman JM, Morey JS, Takeshita R, Guise SD, Wells RS, McFee W, et al. Age determination of common bottlenose dolphins (*Tursiops truncatus*) using dental radiography pulp:tooth area ratio measurements. PLOS ONE. 2020 Nov 20;15(11):e0242273. doi: 10.1371/journal.pone.0242273 33216762 PMC7678971

[51] Mattoon JS, Nyland TG. Small Animal Diagnostic Ultrasound—E-Book. Elsevier Health Sciences; 2014. 687 p.

[52] Forfia PR, Vaidya A, Wiegers SE. Pulmonary heart disease: The heart-lung interaction and its impact on patient phenotypes. Pulm Circ. 2013;3(1):5–19. doi: 10.4103/2045-8932.109910 23662171 PMC3641739

[53] Sommer L, McFarland W, Galliano R, Nagel E, Morgane P. Hemodynamic and coronary angiographic studies in the bottlenose dolphin (*Tursiops truncatus*). Am J Physiol-Legacy Content. 1968 Dec 1;215(6):1498–505. doi: 10.1152/ajplegacy.1968.215.6.1498 4881232

[54] Galiè N, Humbert M, Vachiery J-L, Gibbs S, Lang I, Torbicki A, et al. 2015 ESC/ERS Guidelines for the diagnosis and treatment of pulmonary hypertension: The Joint Task Force for the Diagnosis and Treatment of Pulmonary Hypertension of the European Society of Cardiology (ESC) and the European Respiratory Society (ERS)Endorsed by: Association for European Paediatric and Congenital Cardiology (AEPC), International Society for Heart and Lung Transplantation (ISHLT). Eur Respir J. 2015 Oct 1;46(4):903–75. doi: 10.1183/13993003.01032-2015 26318161

[55] Turner RE, Rabalais NN, Overton EB, Meyer BM, McClenachan G, Swenson EM, et al. Oiling of the continental shelf and coastal marshes over eight years after the 2010 *Deepwater Horizon* oil spill. Environ Pollut. 2019 Sep 1;252:1367–76. doi: 10.1016/j.envpol.2019.05.134 31254894

[56] Michel J, Owens EH, Zengel S, Graham A, Nixon Z, Allard T, et al. Extent and degree of shoreline oiling: *Deepwater Horizon* oil spill, Gulf of Mexico, USA. PLOS ONE. 2013;8(6):e65087. doi: 10.1371/journal.pone.0065087 23776444 PMC3680451

[57] Rouhani S, Baker MC, Steinhoff M, Zhang M, Oehrig J, Zelo IJ, et al. Nearshore exposure to *Deepwater Horizon* oil. Marine Ecology Progress Series. 2017 Aug 3;576:111–24.

[58] Mate BR, Rossbach KA, Nieukirk SL, Wells RS, Irvine AB, Scott MD, et al. Satellite-monitored movements and dive behavior of a bottlenose dolphin (*Tursiops truncatus*) in Tampa Bay, Florida. Mar Mamm Sci. 1995;11(4):452–63.

[59] Mead JG, Potter CW. Recognizing two populations off the bottlenose dolphin (*Tursiops truncatus*) of the Atlantic coast of North America—morphologic and ecologic considerations. 1995 [cited 2020 Feb 17]; Available from: http://repository.si.edu/xmlui/handle/10088/4735

[60] Hoelzel AR, Potter CW, Best PB. Genetic differentiation between parapatric “nearshore” and “offshore” populations of the bottlenose dolphin. Proc Biol Sci. 1998 Jul 7;265(1402):1177–83. doi: 10.1098/rspb.1998.0416 9699311 PMC1689183

[61] Fahlman A, Jensen F, Tyack P, Wells R. Modeling tissue and blood gas kinetics in coastal and offshore common bottlenose dolphins, *Tursiops truncatus*. Front in Physiol. 2018 Jul 17;9. doi: 10.3389/fphys.2018.00838 30072907 PMC6060447

[62] Klatsky LJ, Wells RS, Sweeney JC. Offshore bottlenose dolphins (*Tursiops truncatus*): movement and dive behavior near the Bermuda pedestal. J Mammal. 2007 Feb 28;88(1):59–66.

[63] Wells RS, McHugh KA, Douglas DC, Shippee S, McCabe EB, Barros NB, et al. Evaluation of potential protective factors against metabolic syndrome in bottlenose dolphins: feeding and activity patterns of dolphins in sarasota bay, Florida. Front Endocrinol (Lausanne). 2013 Oct 10;4:139. doi: 10.3389/fendo.2013.00139 24133483 PMC3794307

[64] Wells R, Schwacke L, Rowles T, Balmer B, Zolman E, Speakman T, et al. Ranging patterns of common bottlenose dolphins (*Tursiops truncatus*) in Barataria Bay, Louisiana, following the *Deepwater Horizon* oil spill. Endanger Spec Res. 2017 Jan 1;33.

[65] Wasfy MM, Weiner RB, Wang F, Berkstresser B, Lewis GD, DeLuca JR, et al. Endurance exercise-induced cardiac remodeling: not all sports are created equal. J Am Soc Echocardiogr. 2015 Dec;28(12):1434–40. doi: 10.1016/j.echo.2015.08.002 26361851

[66] Pluim Babette M., Zwinderman Aeilko H., van der Laarse Arnoud, van der Wall Ernst E. The athlete’s heart. Circulation. 2000 Jan 25;101(3):336–44. doi: 10.1161/01.cir.101.3.336 10645932

[67] Fulghum K, Hill BG. Metabolic mechanisms of exercise-induced cardiac remodeling. Front Cardiovasc Med. 2018 Sep 11 [cited 2020 Feb 17];5. Available from: https://www.ncbi.nlm.nih.gov/pmc/articles/PMC6141631/ doi: 10.3389/fcvm.2018.00127 30255026 PMC6141631

[68] Currie KD, Coates AM, Slysz JT, Aubry RL, Whinton AK, Mountjoy ML, et al. Left ventricular structure and function in elite swimmers and runners. Front Physiol. 2018 [cited 2020 Feb 17];9. Available from: https://www.frontiersin.org/articles/10.3389/fphys.2018.01700/full 30546320 10.3389/fphys.2018.01700 PMC6279850

[69] Batista JS, Câmara ACL, Almeida RD, Olinda RG, Silva TMF, Soto-Blanco B. Poisoning by crude oil in sheep and goats. Revue Méd Vét. 2013;5.

[70] Mostrom MS, Campbell C a. J. 1994 livestock field investigation of two ranches associated with a pipeline break. 1998 Nov 1 [cited 2020 Feb 17]; Available from: http://inis.iaea.org/Search/search.aspx?orig_q=RN:30018883

[71] Raekallio M. Long term ECG recording with Holter monitoring in clinically healthy horses. Acta Vet Scand. 1992;33(1):71–5. doi: 10.1186/BF03546937 1598859 PMC8117866

[72] Cw S, Jh R, Mm S van O-O. Continuous monitoring of ECG in horses at rest and during exercise. Vet Rec. 1995 Oct 1;137(15):371–4. doi: 10.1136/vr.137.15.371 8578649

[73] van Loon G, Patteson M. Electrophysiology and arrhythmogenesis. In: Cardiology of the Horse. Elsevier Saunders; 2010 [cited 2021 Mar 11]. p. 59–73. Available from: http://hdl.handle.net/1854/LU-2123298

[74] Reef VB. Review Article: Heart murmurs in horses: determining their significance with echocardiography. Equine Vet J. 1995;27(S19):71–80. doi: 10.1111/j.2042-3306.1995.tb04992.x 8933072

[75] Allen KJ, Erck‐Westergren E van, Franklin SH. Exercise testing in the equine athlete. Equine Vet Educ. 2016;28(2):89–98.

[76] Linnehan BK, Le-Bert CR, McClain AM, Hsu A, Ross KP, Gomez FM, et al. Evaluation of high grade atrioventricular block in a geriatric bottlenose dolphin (*Tursiops runcates*). Proc IAAAM Virtual Conf. 2021 May 23–26. Available from: https://www.vin.com/Proceedings/Proceedings.aspx?orgID=76&said=1

