# Supplementary figures and images for: Cardiac assessments of bottlenose dolphins (Tursiops truncatus) in the Northern Gulf of Mexico following exposure to Deepwater Horizon oil
Source: PLoS One. 2021 Dec 14;16(12):e0261112. doi: 10.1371/journal.pone.0261112 (PMC8670661; doi:10.1371/journal.pone.0261112)

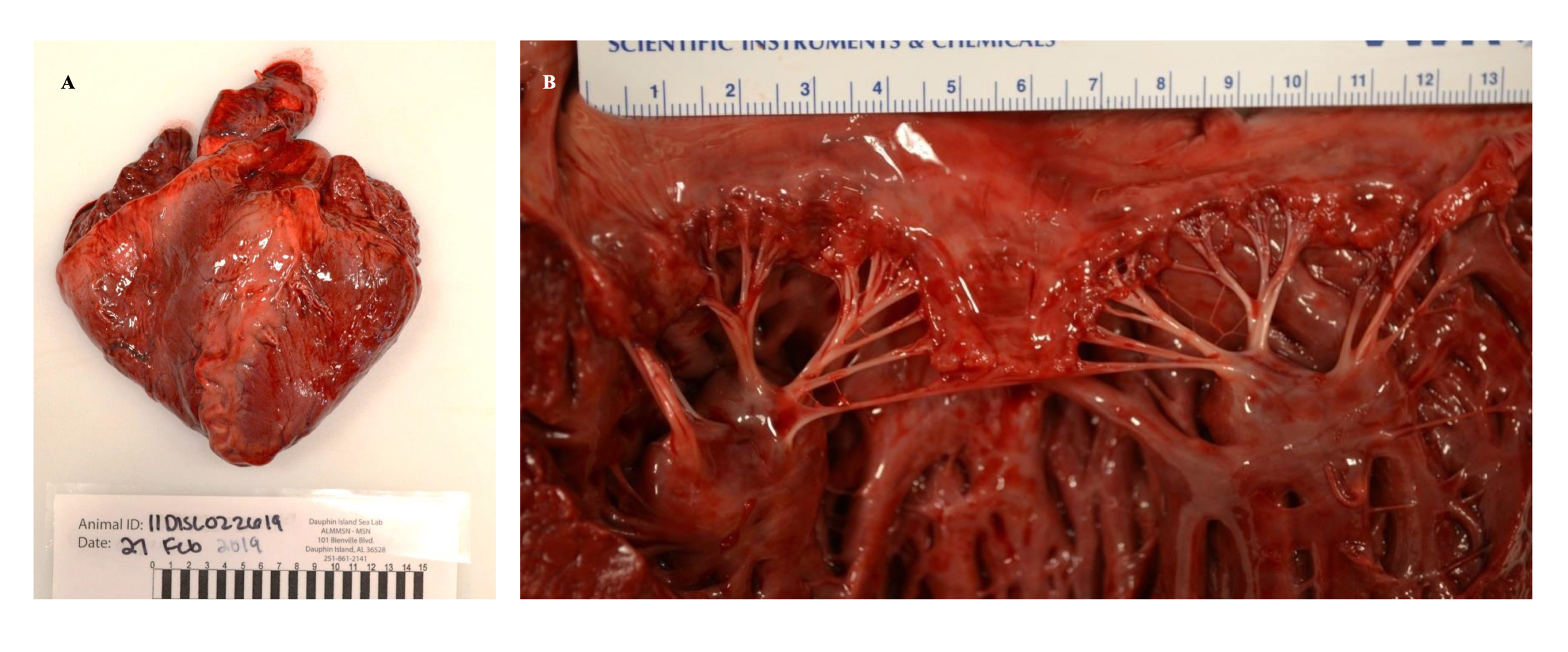

Supplement: S1 Fig — (A) Note the thin right ventricle and pale streaking throughout the epicardium. (B) There is pale tan streaking of the left ventricular endocardial surface and nodular thickening of the leaflets of the mitral valve (endocardiosis). (TIF) [file pone.0261112.s001.tif]
